# Histidine Residue 94 Is Involved in pH Sensing by Histidine Kinase ArsS of *Helicobacter pylori*

**DOI:** 10.1371/journal.pone.0006930

**Published:** 2009-09-07

**Authors:** Stefanie Müller, Monika Götz, Dagmar Beier

**Affiliations:** Theodor-Boveri-Institut für Biowissenschaften, Lehrstuhl für Mikrobiologie, Universität Würzburg, Am Hubland, Würzburg, Germany; University of Hyderabad, India

## Abstract

**Background:**

The ArsRS two-component system is the master regulator of acid adaptation in the human gastric pathogen *Helicobacter pylori*. Low pH is supposed to trigger the autophosphorylation of the histidine kinase ArsS and the subsequent transfer of the phosphoryl group to its cognate response regulator ArsR which then acts as an activator or repressor of pH-responsive genes. Orthologs of the ArsRS two-component system are also present in *H. pylori*'s close relatives *H. hepaticus*, *Campylobacter jejuni* and *Wolinella succinogenes* which are non-gastric colonizers.

**Methodology/Principal Findings:**

In order to investigate the mechanism of acid perception by ArsS, derivatives of *H. pylori* 26695 expressing ArsS proteins with substitutions of the histidine residues present in its periplasmic input domain were constructed. Analysis of pH-responsive transcription of selected ArsRS target genes in these mutants revealed that H94 is relevant for pH sensing, however, our data indicate that protonatable amino acids other than histidine contribute substantially to acid perception by ArsS. By the construction and analysis of *H. pylori* mutants carrying *arsS* allels from the related ε-proteobacteria we demonstrate that WS1818 of *W. succinogenes* efficiently responds to acidic pH.

**Conclusions/Significance:**

We show that H94 in the input domain of ArsS is crucial for acid perception in *H. pylori* 26695. In addition our data suggest that ArsS is able to adopt different conformations depending on the degree of protonation of acidic amino acids in the input domain. This might result in different activation states of the histidine kinase allowing a gradual transcriptional response to low pH conditions. Although retaining considerable similarity to ArsS the orthologous proteins of *H. hepaticus* and *C. jejuni* may have evolved to sensors of a different environmental stimulus in accordance with the non gastric habitat of these bacteria.

## Introduction

The human pathogen *Helicobacter pylori* thrives in the mucous layer covering the gastric epithelium. The neutralophilic bacterium has developed efficient mechanisms to cope with both the high acidity encountered during the passage of the stomach lumen in the initial phase of colonization and moderately acidic conditions expected to prevail in the mucous layer. Central to the acid adaptation of *H. pylori* is the urease system which is essential for maintaining both the cytoplasmic and periplasmic pH near neutrality when the bacteria are exposed to low pH [1]. The cytoplasmic urease enzyme is a nickel-containing dodecameric heterodimer consisting of the subunits UreA and UreB [2] which cleaves urea present in the gastric juice in millimolar concentrations to ammonia and carbon dioxide. Ammonia then acts as a buffering compound in both the cytoplasm and the periplasm. Moreover, carbon dioxide which rapidly diffuses to the periplasmic space is converted to HCO_3_^-^ by the periplasmic α-carbonic anhydrase providing an additional buffering compound [3]. The enzymatic activity of the cytoplasmic urease is controlled by the inner membrane pH-gated channel UreI, which regulates the access of the substrate urea to the bacterial cell in response to acidic pH [4], [5]. Both urease and the channel protein UreI are essential for colonization in several animal infection models [6]–[8]. Furthermore, urease-independent mechanisms of pH-homeostasis are likely to exist [9], [10]. Accordingly, global transcriptional profiling performed by several research groups revealed the differential expression of 100 to about 280 genes in response to the exposure of *H. pylori* to low pH [11]–[14].

The ArsRS two-component system is the master regulator of *H. pylori's* intricate acid response. Acidic pH triggers the autophosphorylation of the histidine kinase ArsS and the subsequent phosphorylation of its cognate response regulator ArsR. Phosphorylated ArsR (ArsR∼P) then acts both as an activator and repressor of pH-responsive genes [14]. The ArsR∼P regulon comprises the urease genes, the amidase genes *amiE* and *amiF*, hp1186 encoding a periplasmic α-carbonic anhydrase, as well as genes encoding antioxidant systems, Ni^2+^-storage proteins, proteins affecting the composition of the cell envelope and *H. pylori*-specific proteins of unknown function [14]–[17]. Consistent with a prominent role of the ArsRS two-component system in the transcriptional control of the acid response, *arsS* null mutants of *H. pylori* were unable to colonize in a mouse infection model [18]. The metal dependent regulators Fur and NikR also contribute to pH-responsive gene regulation, since Fur- and NikR-deficient mutants showed an aberrant transcription profile upon exposure of *H. pylori* to low pH [13], [19]. Furthermore, it was reported that in the *H. pylori* strain J99 the two-component system CrdRS (HP1365-HP1364) which positively regulates the expression of the copper resistance determinant CrdAB-CzcAB in response to increasing concentrations of copper ions [20] is also involved in the pH-responsive regulation of major acid-resistance determinants including the urease gene cluster [21]. This regulatory effect was not observed when CrdR-deficient mutants of the *H. pylori* strains 26695 and G27 were analysed [22]. Recently, the histidine kinase HP0244 which governs the expression of flagellar class II genes was also implicated in pH-responsive transcriptional control [23], [24]. However, the ratios of differential expression were modest in an hp0244 negative mutant and differential expression of most target genes including several members of the ArsR∼P regulon was detected only at extremely low pH [24].

In this study we investigated the mechanisms by which the sensor protein ArsS perceives acidic pH. It was assumed that protonation of specific amino acid residues in the periplasmic input domain of ArsS eliciting a conformational change of the histidine kinase is involved in pH sensing. Furthermore, we analysed the ability of ArsS orthologs from other members of the ε-proteobacteria to respond to acidic pH.

## Materials and Methods

### Bacterial strains and growth conditions

*H. pylori* 26695 and G27 are clinical isolates which have been described previously [25], [26]. *H. pylori* strains were grown at 37°C under microaerophilic conditions (Oxoid) on Columbia agar plates containing 5% horse blood, 0.01% cycloheximide and Skirrow's antibiotic supplement. Liquid cultures were grown in brain heart infusion (BHI) broth containing Skirrow's antibiotic supplement and 10% fetal calf serum (FCS). When required blood agar plates or liquid broth for *H. pylori* culture were supplemented with kanamycin or chloramphenicol in a final concentration of 20 µg/ml. Acid exposure experiments were performed as follows: Bacteria from a liquid culture were harvested at an OD_590_ of 0.7 by centrifugation and were then shifted for one hour to supplemented BHI broth whose pH had been adjusted to pH 5.0 with hydrochloric acid. In case of neutral pH controls cultivation was continued for one hour in standard BHI broth. *E. coli* DH5α was grown in Luria-Bertani (LB) broth which was supplemented with antibiotics in the following final concentrations when necessary: ampicillin 100 µg/ml, kanamycin 50 µg/ml, chloramphenicol 30 µg/ml.

### Construction of *H. pylori* strains carrying mutated alleles of *arsS*

To construct derivatives of *H. pylori* 26695 expressing mutated ArsS proteins a two step allelic exchange procedure was applied. Natural transformation or electroporation of *H. pylori* strains was performed essentially as described previously [27], [28]. First in *H. pylori* 26695 the wild-type *arsS* gene was replaced by a kanamycin resistance cassette via transformation with suicide plasmid pSL-165::km [29]. The resulting *arsS* null mutant 26695/arsS::km was then transformed with suicide plasmids comprising the mutated *arsS* allele and a *cat* gene from *C. coli* [30] flanked by DNA fragments derived from *arsR* and the *hemB* (hp0163) gene. Allelic exchange resulted in the substitution of the *aphA* gene by both the respective *arsS* allele and the *cat* gene. The suicide plasmids were derived from a precursor construct, pSL-arsSTD, which contains a EcoRI-SmaI fragment encoding amino acids (aa) 97–225 of ArsR (PCR amplified with primer pair arsR-5/arsR-3, Table 1), a BglII-XbaI fragment encoding aa 163–428 of ArsS (PCR amplified with primer pair arsSTD-5/arsSTD-3), a XbaI-PstI fragment containing the chloramphenicol resistance cassette, and a PstI-SacI fragment (PCR amplified with primer pair 0163-5/0163-3) encoding aa 1–188 of HP0163 (HemB, δ-aminolevulinic acid dehydratase). A SmaI-BglII fragment encoding aa 1–162 of ArsS (PCR amplified with primer pair arsSID-5/arsSID-3) was cloned into pSL1180 yielding the template for the generation of mutated *arsS* alleles via recombinant PCR [31]. To make sure that the acid response of *H. pylori* 26695 was not altered by the insertion of the *cat* gene into the hp0166-hp0162 operon or minor sequence modifications introduced for cloning purposes, a control strain (26695/arsS-H0) was constructed carrying wild-type *arsS* flanked by the *cat* gene. For the construction of double mutants a derivative of pSL-arsSID was used whose SmaI-BglII insert encodes an ArsS input domain with a H94A substitution. Finally, the SmaI-BglII fragments containing the desired point mutations were cloned into plasmid pSL-arsSTD. All cloned PCR fragments which were amplified from chromosomal DNA of *H. pylori* 26695 using the primers listed in Table 1 or were obtained by recombinant PCR performed on plasmid pSL-arsSID were subjected to automated sequencing to ensure proper PCR amplification. Transformants obtained by allelic exchange mutagenesis of *H. pylori* 26695/arsS::km were checked for the correct integration of the mutated *arsS* alleles by PCR analysis with primer pairs flanking the integration site.

**Table 1:** Oligonucleotides used in this study.

Name	Sequence (5′ to 3′)^a^	Site^b^	Strand	Position^c^
arsR-5	tatggg gaattc GATTACCTCCCTAAACCCTATG	EcoRI	−	174146–174167
arsR-3	ctcctt cccggg ATATCAGTATTCTAATTTATAA	SmaI	+	173775–173796
0163-5	aatttg ctgcag GTGAGCGGAATGAAGGGGATAG	PstI	−	172443–172464
0163-3	agtgga gagctc CATGATGGGCGTGTGGGTATAG	SacI	+	171835–171856
arsSID-5	gaatac cccggg TCTAATAAGGAGTTAAGGGGTT	SmaI	−	173753–173774
arsSID-3	cacttg AGATCT TAACTCTCTTAAGGGCAATAAA	BglII	+	173267–173294
arsSTD-5	gagtta AGATCT CAAGTGAAACGCTTCGCTCAAG	BglII	−	173246–173272
arsSTD-3	cccttc tctaga CTCACTTCTCTCAAATTTTCG	XbaI	+	172460–172480
HH1607-5	aaattt cccggg aaggaGAGATTCTATGTTTCAATTTCG	SmaI	−	1535356–1535377
HH1607-3	aaattt tctaga CATAGATTCTCCTCTATTTTG	XbaI	+	1534096–1534117
CJ1262-5	aaattt cccggg aaggagTGATAGGATGACAAAAAATTA	SmaI	+	1192449–1192469
CJ1262-3	aaattt ctgcag CAGGCCTTTTACCATTATTTT	PstI	−	1193685–1193705
WS1818-5	aaattt cccggg aaggagCTCGACCATGACTAAAAACTC	SmaI	+	1712553–1712573
WS1818-3	aaattt tctaga GAGAGAAAAGCCTCACTTCTC	XbaI	−	1713781–1713801
HH1607-E5	ttgact ggatcC AAAACACCAAAGATGAAATTGGGGAA	BamHI	−	1534803–1534829
HH1607-E3	ctctct gaattc CTATTTTGGTTTTTTATCAACAAG	EcoRI	+	1534110–1534133
HH1608-E5	ttcact ggatcc TTAGAAGTTTTAATGATTGAAGATG	BamHI	−	1536037–1536061
HH1608-E3	aaaaaa ctgcag TTATGTTTCAAGTTTATAGCCCACA	PstI	+	1535384–1535408
CJ1262-E5	tttcta ggatcc AAGAAGATGAAGTAGGCAAGA	BamHI	+	1193003–1193023
CJ1262-E3	cctttt gaattc TTACCATTATTTTTCTTTATCTCCA	EcoRI	−	1193673–1193697
CJ1261-E5	acacta ggatcc ATTAATGTGTTGATGATAGAAGATG	BamHI	+	1191791–1191815
CJ1261-E3	aataat ctgcag TCATCCTATCAGTTTATATCCTATA	PstI	−	1192435–1192459
WS1818-E5	ttcact ggatcC AAAACGATGAGATTGGTGAGC	BamHI	+	1713094–1713115
WS1818-E3	ctctct gaattc TCACTTCTCAACCACAACGCC	EcoRI	−	1713769–1713789
WS1817-E5	ttcact ggatcc CTAGAGATTCTGATGATTGAGG	BamHI	+	1711878–1711899
WS1817-E3	aaaaa ctgcag GTGCCACAGCAAAGAGAATCG	PstI	−	1712591–1712611
HP119PE	TCATCATCATTGTTGCAAGC		+	130723–130742
HP1432PE	CCGTAGTAATGGTGGTGGTGCG		−	1502650–1502671

a Sequences in upper case letters are derived from the genome sequences of *H. pylori* 26695 [25], *H. hepaticus* [32], *C. jejuni* [33] and *W. succinogenes* [34]. Sequences introduced for cloning purposes are given in lower case letters, restriction recognition sequences are underlined.

b Restriction recognition sites.

c Nucleotide positions refer to the genome sequence of *H. pylori* 26695 [25], *H. hepaticus* [32], *C. jejuni* [33] and *W. succinogenes* [34].

### Construction of *H. pylori* strains with substitutions of *arsS* by the orthologous genes of *H. hepaticus*, *C. jejuni* and *W. succinogenes*

The *arsS* orthologs HH1607 from *H. hepaticus* ATCC51449 [32], CJ1262 from *C. jejuni* 4344 [33] and WS1818 from *W. succinogenes* DSMZ1740 [34] were PCR-amplified from chromosomal DNA of the respective strain using primer pairs HH1607-5/HH1607-3, CJ1262-5/CJ1262-3 and WS1818-5/WS1818-3, respectively. The resulting DNA-fragments encoding HH1607, CJ1262 and WS1818, respectively, were ligated into plasmid pSL1180 containing the 393 bp EcoRI-SmaI fragment derived from *arsR*, the chloramphenicol resistance cassette from *C. coli* [30], and the 630 bp PstI-SacI fragment derived from *hemB* yielding suicide plasmids pSL-HH1607 cm, pSL-CJ1262 cm and pSL-WS1818 cm. These suicide plasmids were used for the transformation of *H. pylori* G27/HP165::km which carries a substitution of *arsS* by a kanamycin resistance cassette [29]. Selection for chloramphenicol resistance resulted in the replacement of the *aphA* gene by the respective *arsS* ortholog which was checked by PCR analysis of chromosomal DNA of the transformants.

### RNA isolation and primer extension analysis

RNA from *H. pylori* was isolated from bacteria grown to the logarithmic phase in liquid broth by using TRIzol reagent (Invitrogen) according to the manufacturer's protocol. The RNA concentration was quantified by determination of the absorbance at 260 and 280 nm, and RNA integrity was checked by visualization on a 1.5% agarose gel. Primer extension analysis was performed essentially as described previously [35] using 0.5 pMol of γ^32^P-end-labelled oligonucleotides HP1432PE and HP119PE, respectively. Primer extension experiments were performed three times on two independently prepared RNAs. Quantification of the signals from the primer extension products was performed using a Typhoon 9200 Variable Mode Imager (Amersham Biosciences) and ImageMaster TotalLab Software (Amersham Biosciences).

### Construction of plasmids expressing recombinant ArsS and ArsR orthologs from *H. hepaticus*, *C. jejuni* and *W. succinogenes* and purification of fusion proteins

DNA fragments encoding the transmitter domains of histidine kinase HH1607 from *H. hepaticus* ATCC51449 (aa 181–419), CJ1262 from *C. jejuni* 4344 (aa 185–411) and WS1818 from *W. succinogenes* DSMZ1740 (aa 179–409) were PCR-amplified from chromosomal DNA of the respective strain using primer pairs HH1607-E5/HH1607-E3, CJ1262-E5/CJ1262-E3 and WS1818-E5/WS1818-E3. The DNA fragments were ligated into BamHI and EcoRI-digested pGEX-3X vector DNA generating in frame-fusions of the vector-borne glutathione S-transferase (*gst*) gene and the ORFs encoding the transmitter domains of HH1607, CJ1262 and WS1818, respectively. The *arsR* orthologs hh1608, cj1261 and ws1817 were PCR-amplified with primer pairs HH1608-E5/HH1608-E3, CJ1261-E5/CJ1261-E3 and WS1817-E5/WS1817-E3, respectively, and the resulting BamHI-PstI fragments were cloned into pQE30 vector DNA creating N-terminal His_6_-tags. Plasmid pGEX-165 encoding a fusion of the transmitter domain of ArsS to glutathione S-transferase and plasmid pQE-166 encoding ArsR fused to an N-terminal His_6_-tag have been described earlier [29].

Fusion proteins GST-ArsS, GST-HH1607, GST-CJ1262 and GST-WS1818 were produced in *E. coli* DH5α and were purified by affinity chromatography on glutathione-Sepharose 4B as described previously [29]. The His_6_-tagged response regulator proteins were overproduced in *E. coli* M15 (pREP4) (Qiagen) and protein purification on Ni^2+^-nitrilotriacetic acid agarose columns was carried out as described [29].

### In vitro phosphorylation assays

In vitro phosphorylation assays providing multiple turnover conditions were performed in a final volume of 25 µl of reaction buffer (50 mM Tris-HCl, pH 7.5, 50 mM KCl, 10 mM MgCl_2_, 0.5 mM ATP, 420 nM [γ^33^P]ATP (3000 Ci/mmol)). The reaction mixtures were incubated at room temperature for 10 min. The reactions were stopped by the addition of sample buffer (60 mM Tris-HCl, pH 7.5, 50 mM Na_2_EDTA, 10% glycerol, 2% SDS, 5% β-mercaptoethanol, 0.05% bromophenol blue) and the reaction mixtures were separated by electrophoresis on a 12% SDS-polyacrylamide gel (1.5 mm).

## Results and Discussion

### H94 in the periplasmic input domain of ArsS is involved in low pH-sensing

We hypothesized that signal perception by the ArsS sensor protein requires protonation of amino acids in the periplasmic input domain which triggers a conformational change of the protein stimulating its histidine kinase activity. Differential expression of pH-responsive genes was observed when *H. pylori* is shifted from neutral pH to pH 5.0 [11]–[14]. Histidine seemed to be best suited to participate in pH perception by ArsS, since according to its side chain *pK_a_* (6.1) free histidine residues will be protonated when the pH drops below 6, while the side chains of arginine (*pK_a_* = 12,5) and lysine (*pK_a_* = 10.8) are already protonated at neutral pH. The side chain carboxyl groups of glutamate and aspartate will change their protonation state at a pH around 4.0 (*pK_a_* = 4.3 and 3.9, respectively). Previously, protonation of histidine residues in the second periplasmic loop and the periplasmic C-terminus of the pH-gated urea channel UreI of *H. pylori* was shown to be required for the low pH activation of urea transport [36]. Furthermore, a histidine residue in the periplasmic input domain of histidine kinase PmrB from *Salmonella enterica* was reported to be involved in pH-responsive activation of the PmrAB two-component system [37].

**Figure 1:**
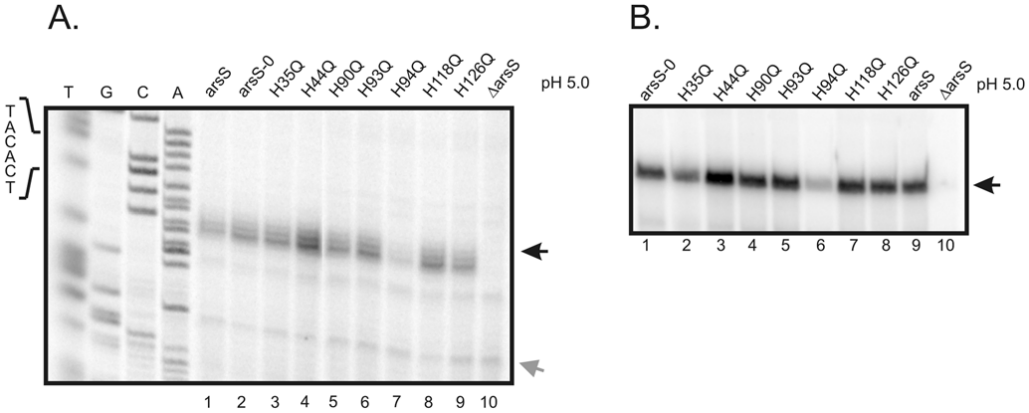
Analysis of transcription of the acid-responsive genes hp0119 (A.) and hp1432 (B.) in *H. pylori* strains expressing ArsS proteins with individual histidine to glutamine mutations in the input domain. A. Primer extension analysis using the radiolabelled oligonucleotide HP119PE was performed on equal amounts of RNA extracted from the *H. pylori* strains 26695 (lane 1), 26695/arsS-H0 (lane 2), 26695/arsS-H35Q (lane 3), 26695/arsS-H44Q (lane 4), 26695/arsS-H90Q (lane 5), 26695/arsS-H93Q (lane 6), 26695/arsS-H94Q (lane 7), 26695/arsS-H118Q (lane 8), 26695/arsS-H126Q (lane 9) and 26695/arsS::km (lane 10) which were exposed to pH 5.0 for one hour. The hp0119-specific cDNA is indicated by a black arrow on the right. The sequencing ladder (lanes T, G, C, A) was obtained by annealing oligonucleotide HP119PE to plasmid pSL-119PE2 [39]. The gray arrow indicates the position of a cDNA band representing a non-specific primer elongation product whose unchanged intensity indicates the presence of equal amounts of RNA in the different samples. The sequence of the -10 element of the hp0119 promoter is shown on the left. B. Primer extension analysis using the radiolabelled oligonucleotide HP1432PE was performed on equal amounts of RNA extracted from the *H. pylori* strains 26695 (lane 9), 26695/arsS-H0 (lane 1), 26695/arsS-H35Q (lane 2), 26695/arsS-H44Q (lane 3), 26695/arsS-H90Q (lane 4), 26695/arsS-H93Q (lane 5), 26695/arsS-H94Q (lane 6), 26695/arsS-H118Q (lane 7), 26695/arsS-H126Q (lane 8) and 26695/arsS::km (lane 10) which were exposed to pH 5.0 for one hour. The hp1432-specific cDNA is indicated by a black arrow on the right.

**Figure 2:**
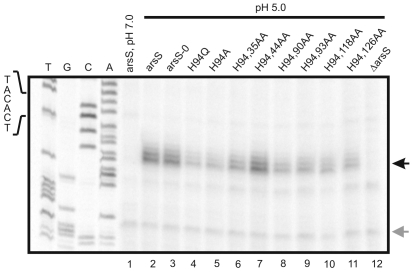
Analysis of transcription of the acid-responsive gene hp0119 in *H. pylori* strains expressing ArsS proteins with histidine to alanine double mutations in the input domain. Primer extension analysis using the radiolabelled oligonucleotide HP119PE was performed on equal amounts of RNA extracted from the *H. pylori* strains 26695 (lane 2), 26695/arsS-H0 (lane 3), 26695/arsS-H94Q (lane 4), 26695/arsS-H94A (lane 5), 26695/arsS-H94,35AA (lane 6), 26695/arsS-H94,44AA (lane 7), 26695/arsS-H94,90AA (lane 8), 26695/arsS-H94,93AA (lane 9), 26695/arsS-H94,118AA (lane 10), 26695/arsS-H94,126AA (lane 11) and 26695/arsS::km (lane 12) which were exposed to pH 5.0 for one hour. As a control primer extension analysis was also performed on RNA from the wild-type strain 26695 grown at pH 7.0 (lane 1). The hp0119-specific cDNA is indicated by a black arrow on the right. The sequencing ladder (lanes T, G, C, A) was obtained by annealing oligonucleotide HP119PE to plasmid pSL-119PE2 [39]. The sequence of the -10 element of the hp0119 promoter is shown on the left. The gray arrow indicates the position of a cDNA band representing a non-specific primer elongation product whose unchanged intensity indicates the presence of equal amounts of RNA in the different samples.

The periplasmic domain of ArsS contains seven histidine residues (H35, H44, H90, H93, H94, H118, H126) which are not conserved in the N-terminal domains of the orthologous proteins from the related ε-proteobacteria *H. hepaticus*, *C. jejuni* and *W. succinogenes* [32]–[34]. Derivatives of *H. pylori* 26695 expressing ArsS proteins with individual glutamine substitutions of these seven histidine residues were constructed and pH-responsive transcription of selected members of the ArsR∼P regulon was analysed in the mutants. Target gene hp1432 encodes an Hpn-like Ni^2+^-storage protein. In addition to being controlled by ArsR∼P in response to low pH [15], hp1432 is subject to Ni^2+^-responsive regulation by the regulator protein NikR [38]. In case of hp0119 encoding an *H. pylori*-specific protein of unknown function the direct binding of ArsR∼P to its promoter region has been demonstrated [39]. The 26695 *arsS* mutants as well as the control strain 26695/arsS-H0, the 26695 wild-type and the isogenic *arsS* null mutant were grown to an OD_590_ of 0.7 at neutral pH and were then shifted to pH 5.0 for one hour. Primer extension analysis was performed on equal amounts of RNA extracted from the acid-exposed bacteria using oligonucleotides specific for hp0119 and hp1432. As shown in Fig. 1A acid-responsive transcription of hp0119 in *H. pylori* 26695/arsS-H0 was indistinguishable from the wild-type strain 26695 and no significant alterations were observed in the *H. pylori* 26695 mutants expressing ArsS proteins with individual H35Q, H90Q, H93Q, H118Q and H126Q substitutions, respectively. However, in strain 26695/arsS-H94Q the amount of hp0119-specific transcript was reduced to about one fourth of the wild-type transcript level, while a slight increase in the amount of hp0119-specific transcript was detected in strain 26695/arsS-H44Q. The same results were obtained when pH-responsive transcription of hp1432 was analysed (Fig. 1B). Transcription of target genes at neutral pH was not affected in the mutants (data not shown). These data indicate a role of H94 in the mechanism of pH sensing by the periplasmic input domain of ArsS. To corroborate this conclusion an *H. pylori* mutant expressing a derivative of ArsS with a substitution of H94 to alanine was constructed which also exhibited reduced pH-responsive transcription of hp0119 and hp1432 (Fig. 2 and data not shown).

Since residual pH-responsive transcriptional induction of ORFs hp0119 and hp1432 was still observed in strains 26695/arsS-H94Q and 26695/arsS-H94A (Fig. 1, Fig. 2), double mutants were constructed by replacing H35, H44, H90, H93, H118 and H126, respectively, by alanine in ArsS-H94A. Compared to 26695/arsS-H94A no further decrease in the amounts of hp0119- and hp1432-specific transcripts at pH 5.0 was detected in the *H. pylori* mutants expressing the proteins ArsS-H94,35AA, ArsS-H94,90AA, ArsS-H94,93AA, ArsS-H94,118AA and ArsS-H94,126AA. However, to our surprise mutant 26695/arsS-H94,44AA showed wild-type pH-responsive transcription of hp0119 and hp1432 (Fig. 2 and data not shown). When mutant 26695/arsS-H94,44AA was grown at neutral pH, transcription of hp0119 was not altered compared to the wild-type, while a marginal increase in the amount of hp1432-specific transcript was detected (data not shown). It is known that within a protein the pK*_a_* of its residues can deviate substantially from the pK*_a_* of the free amino acids. Therefore, we hypothesize that the H44A mutation in ArsS-H94,44AA might increase the side chain pK*_a_* of acidic amino acids in the input domain, whose protonation contributes to the activation of ArsS, thereby compensating for the H94A mutation.

**Figure 3:**
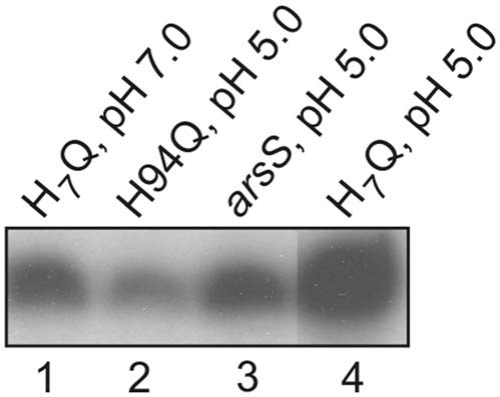
Analysis of transcription of hp0119 in *H. pylori* 26695/arsS-H_7_Q carrying substitutions of the input domain histidines H35, H44, H90, H93, H94, H118 and H126 of ArsS to glutamine. Primer extension analysis using the radiolabelled oligonucleotide HP119PE was performed on equal amounts of RNA extracted from the *H. pylori* strain 26695/arsS-H_7_Q grown at pH 7.0 (lane 1) and strains 26695 (lane 3), 26695/arsS-H94Q (lane 2), and 26695/arsS-H_7_Q (lane 4) exposed to pH 5.0 for one hour.

Surprisingly, concerted substitution of all seven histidine residues in the periplasmic domain of ArsS by glutamine resulted in a *H. pylori* mutant which at neutral pH exhibited transcription of hp0119 and hp1432 at a level similar to that observed in the wild-type strain 26695 at pH 5.0, suggesting that the mutant ArsS protein adopts a locked-on conformation. However, lowering the pH from 7.0 to 5.0 still triggered an about five-fold increase in transcription of the ArsRS target genes analysed (Fig. 3 and data not shown). These data indicate that pH sensing by ArsS does not rely exclusively on the protonation of histidine residues but rather suggest a complex structural interplay of various amino acids changing charge at low pH. In other pH sensing histidine kinases protonation patterns of similar complexity have been proposed to result upon lowering of the pH. In case of PmrB from *S. typhimurium*, in addition to H35, four glutamic acid residues in the periplasmic domain were shown to contribute to acid sensing [37]. Analysis of the sensor domain of the *Salmonella* histidine kinase PhoQ, which responds to low Mg^2+^, acidic pH and antimicrobial peptides, by NMR spectroscopy revealed a low pH-induced conformational change involving multiple amino acid interactions [40]. The input domain of ArsS comprises five aspartic acid and nine glutamic acid residues whose contribution to pH sensing remains to be investigated in detail. Furthermore, our results suggest that depending on the protonation state of the periplasmic input domain ArsS might adopt variable conformations corresponding to different activation states of the histidine kinase. In this context it should be noted that Wen et al. observed a gradual response of the members of the low pH-stimulon of *H. pylori* when the bacteria were shifted from neutral pH to pH 6.2, 5.5 and 4.5, respectively [12], [16].

### The ArsS ortholog WS1818 of *W. succinogenes* responds to acidic pH

**Figure 4:**
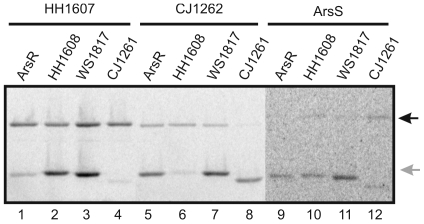
In vitro phosphorylation of response regulator ArsR and its orthologs HH1608, WS1817 and CJ1261 by the histidine kinases ArsS, HH1607 and CJ1262. The recombinant histidine kinases (1 µM) HH1607 (lanes 1–4), CJ1262 (lanes 5–8) and ArsS (lanes 9–12) were incubated in the presence of γ^33^P-ATP and the response regulators (5 µM) ArsR (lanes 1, 5 and 9), HH1608 (lanes 2, 6 and 10), WS1817 (lanes 3, 7, and 11) and CJ1261 (lanes 4, 8, and 12), respectively, for 10 min at room temperature. The reactions were stopped by the addition of sample buffer and the reaction mixtures were separated on a 12% SDS-polyacrylamide gel. The position of the histidine kinases and response regulators is indicated by black and grey arrows on the right.

Although they have to pass through the stomach during infection of their hosts, *H. hepaticus*, *C. jejuni* and *W. succinogenes* are not expected to encounter extended periods of extremely low pH according to their non-gastric habitats and probably do not require acid adaptation systems as sophisticated as the one of *H. pylori*. Consistent with this view *C. jejuni* and *W. succinogenes* do not encode urease [41]. *H. hepaticus* encodes urease, however, the activity of the enzyme is acid-independent and urease is not required for the efficient cecal colonization of mice [42], [43]. Therefore, we asked whether the ArsS orthologs of *H. hepaticus*, *C. jejuni* and *W. succinogenes*, which exhibit 34.6%, 22.3% and 28.5% identity with ArsS in their input domains, are able to sense pH changes. To test this we intended to analyse the acid response of mutant *H. pylori* strains expressing the respective *arsS* ortholog, i.e. HH1607, CJ1262 or WS1818, instead of *arsS*. A premise for this approach is the efficient phosphorylation of response regulator ArsR by the ArsS orthologs. The transmitter domains of the ArsS orthologs and the ArsR orthologs show considerable similarity with each other (% identity/similarity with ArsS: HH1607: 51.4/60.1, CJ1262: 38.1/52.0, WS1818: 45.4/60.1; % identity/similarity with ArsR: HH1608: 72.8/82.6, CJ1261: 52.5/62.4, WS1817: 68.4/77.8). First we performed in vitro phosphorylation assays with the purified recombinant histidine kinase domains of the ArsS orthologs and the cognate response regulator proteins. As shown in Fig. 4 autophosphorylation of histidine kinases ArsS, HH1607 and CJ1262 and phosphoryl group transfer to their cognate response regulators as well as to the three orthologous response regulator proteins could be detected. Autophosphorylation of WS1818 was not observed under our experimental conditions. While cross phosphorylation between the ArsRS orthologs from *H. hepaticus* and *C. jejuni* was weak, ArsR from *H. pylori* was phosphorylated to a considerable extent by histidine kinases HH1607 from *H. hepaticus* and CJ1262 from *C. jejuni*. As expected no phosphorylation of the response regulators was observed when the proteins were incubated with γ^33^P-ATP in the absence of a histidine kinase (data not shown).

**Figure 5:**
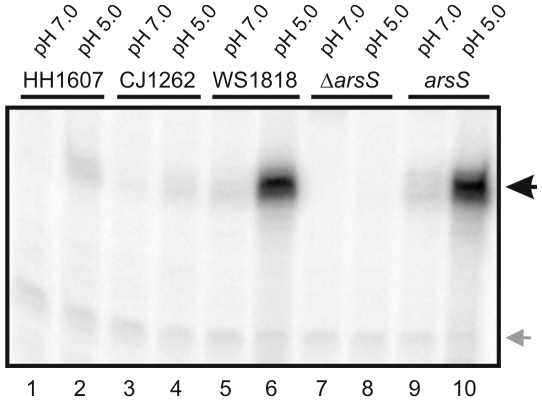
Analysis of the acid-responsive expression of hp0119 in *H. pylori* G27 and mutant *H. pylori* strains expressing the ArsS orthologs of *H. hepaticus*, *C. jejuni* and *W. succinogenes*. Primer extension analysis with a radiolabelled oligonucleotide specific for hp0119 was performed on equal amounts of RNA extracted from strains *H. pylori* G27 (lanes 9 and 10), G27/HP165::km (lanes 7 and 8), G27/HH1607 (lanes 1 and 2), G27/CJ1262 (lanes 3 and 4) and G27/WS1818 (lanes 5 and 6) which were grown at pH 7.0 (lanes 1, 3, 5, 7, and 9) or were exposed to pH 5.0 for one hour (lanes 2, 4, 6, 8, and 10). The hp0119-specific cDNA is indicated by a black arrow on the left. The gray arrow indicates the position of a cDNA band representing a non-specific primer elongation product whose unchanged intensity indicates the presence of equal amounts of RNA in the different samples.

Since the transmitter domains of HH1607, CJ1262 and ArsS proved to be similar enough to allow the phosphorylation of ArsR by the orthologous histidine kinases, mutants of *H. pylori* G27 were constructed which express the histidine kinases HH1607, CJ1262 and WS1818, respectively, instead of ArsS. Strain G27 was chosen for mutant construction because it does not express the restriction/modification system HpyAV [44] whose recognition sites are present in the genes hh1607, cj1262 and ws1818. RNA was prepared from liquid cultures of the wild-type strain *H. pylori* G27, the *arsS* null mutant G27/HP165::km [29] and the hybrid strains G27/HH1607, G27/CJ1262 and G27/WS1818 grown at neutral pH and exposed to pH 5.0 for one hour. Primer extension analysis performed with an hp0119-specific oligonucleotide revealed similar transcript amounts in the G27 wild-type and the hybrid strain G27/WS1818 grown at pH 5.0 indicating that an efficient acid response is triggered by WS1818 upon pH downshift. In case of G27/HH1607 a slight increase in the transcription of hp0119 was observed upon exposure to pH 5.0, while similar amounts of transcript were detected in RNA extracted from G27/CJ1262 at neutral and acidic pH. However, transcription of hp0119 was more pronounced in G27/CJ1262 than in the *arsS* null mutant G27/HP165::km indicating that histidine kinase CJ1262 is expressed in *H. pylori* and causes low-level phosphorylation of ArsR (Fig. 5). These data demonstrate that histidine kinase WS1818 is indeed an acid sensor and suggest that HH1607 and CJ1262 might perceive other stimuli than low pH. At present it cannot be ruled out that the observed lack of acid responsiveness of HH1607 and CJ1262 in the genetic background of *H. pylori* is due to inefficient expression of the histidine kinases. So far attempts to raise an ArsS-specific polyclonal antibody suitable for proving the production of the ArsS orthologs in *H. pylori* have failed. However, in this context it should be noted that a response regulator protein of *C. jejuni* has been successfully expressed in *H. pylori* [45]. It is also conceivable that WS1818 or even ArsS recognize other stimuli in addition to low pH as has been demonstrated for other histidine kinases like *Salmonella* PhoQ [46]. Interestingly, the input domain of WS1818 harbours a histidine residue at position 92 corresponding to H94 in ArsS. However, substitution of H92 by glutamine did not compromise the acid-responsive transcription of hp0119 in G27/WS1818 (data not shown).

In conclusion, in this study we present evidence that H94 in the periplasmic input domain is involved in the perception of low pH by histidine kinase ArsS. However, a comprehensive mutation analysis of all the histidine residues in the input domain indicated that additional parts of the protein contribute substantially to acid sensing. Future experiments will aim at the elucidation of the role of acidic amino acids of the input domain in the mechanism of acid sensing by ArsS. Moreover we showed that the histidine kinase WS1818 of *W. succinogenes* is able to functionally complement an *arsS* deficient mutant of *H. pylori*.
